# Cage-Farming Causes Histopathological Alterations in the Renal Tissues of the Rainbow Trout *Oncorhynchus mykiss* (Walbaum, 1792)

**DOI:** 10.3390/ijms262210876

**Published:** 2025-11-09

**Authors:** Marina Ugrin, María Fernandez Godoy, Ivana Restović, Jerko Hrabar, Nives Kević, Ivana Bočina

**Affiliations:** 1 Doctoral Study Program in Biophysics, Faculty of Science, University of Split, Ruđera Boškovića 33, 21000 Split, Croatia; mugrin4@pmfst.hr; 2 Faculty of Sciences, University of Málaga, Blvr. Louis Pasteur 31, Puerto de la Torre, 29010 Málaga, Spain; maria.fgodoy17@gmail.com; 3 Faculty of Humanities and Social Studies, University of Split, Poljička Cesta 35, 21000 Split, Croatia; irestovic@ffst.hr; 4 Institute of Oceanography and Fisheries, Šetalište Ivana Meštrovića 63, 21000 Split, Croatia; hrabar@izor.hr; 5 Department of Biology, Faculty of Science, University of Split, Ruđera Boškovića 33, 21000 Split, Croatia; bocina@pmfst.hr

**Keywords:** rainbow trout, kidney, cage-farming, TEM, immunofluorescence

## Abstract

Fish are widely recognized as effective bioindicators in ecotoxicological studies due to their repeated exposure to aquatic pollutants that accumulate in metabolically active organs, often leading to histopathological changes. In aquaculture, cage-farmed fish experience continuous environmental and culture-related stress, which can affect renal integrity. The kidney, a central osmoregulatory organ, is particularly sensitive to such conditions. Renal tissues were collected from different growth stages of cage-farmed rainbow trout. Hematoxylin and eosin staining was performed to detect morphological alterations, while transmission electron microscopy was used to assess cellular damage at the ultrastructural level. The expression of fibronectin and caspase-3, markers of extracellular matrix remodeling and apoptosis, respectively, was also evaluated. TEM examination showed pronounced alterations in both the glomeruli and renal tubules, accompanied by increased expression of fibronectin and caspase-3, indicating ongoing tissue remodeling and cellular stress. This study demonstrates that cage-farmed rainbow trout exhibit progressive ultrastructural kidney alterations that appear to be associated with environmental confinement, nutritional practices, and prophylactic treatments. These conditions collectively contribute to renal stress and the onset of nephropathic changes in aquaculture settings. Further research should focus on molecular marker expression to better understand renal adaptation and injury progression under intensive farming conditions.

## 1. Introduction

Aquaculture represents one of the fastest-growing food-producing sectors worldwide, providing over half of the fish consumed by humans [1]. It plays an essential role in global food security as natural fish resources can no longer meet the growing demand for seafood [2]. Intensive aquaculture systems, including ponds, recirculating systems, and open-water cages, allow high productivity but also expose fish to environmental and physiological stressors such as crowding, unstable oxygen levels, and variable water quality [3,4]. Among these, cage aquaculture has become increasingly common in lakes and coastal areas due to its efficiency and suitability, yet it subjects fish to natural environmental fluctuations, pollutants, and pathogen exposure [5,6]. Such chronic stress conditions can impair immunity, metabolism, and organ function, leading to histopathological alterations in vital tissues such as the liver, gills, and kidney [7]. Therefore, understanding the physiological and molecular effects of intensive, cage-based aquaculture systems is essential for improving fish welfare and ensuring sustainable production practices.

Fish, commonly used as bioindicators in ecotoxicology, are particularly sensitive to environmental pollutants due to their aquatic lifestyle [8]. In cage-farmed fish, specific environmental and feeding conditions influence their susceptibility to kidney [9]. Unlike wild fish, cage-farmed fish are fed artificial diets designed to promote commercial growth and are confined to limited spaces, making them vulnerable to infections and environmental stressors [10,11]. To mitigate these risks, preventive treatments, including antibiotics and chemicals, can aggravate kidney damage in these fish [12].

In fish, the kidney plays an important role in maintaining electrolyte and water balance, ensuring a stable internal environment [13]. Exposure to various environmental contaminants is known to induce histopathological alterations in fish, which are continuously used as biomarkers for assessing aquatic organism health [7,8,14]. Among aquatic vertebrates, toxicants primarily enter the body through the gills, subsequently disrupting the histological architecture of vital metabolic organs such as the liver and kidney [12]. Early detection, management, and prevention of renal dysfunction requires the identification and application of reliable markers indicative of kidney damage [15,16].

Comparative studies show that renal responses to aquaculture stressors vary among species. For example, common carp (*Cyprinus carpio*) exhibit glomerular and tubular degeneration when exposed to pesticides such as deltamethrin [17], while Nile tilapia (*Oreochromis niloticus*) show tubular necrosis and congestion following chronic ammonia exposure [18]. Similarly, antibiotic-treated tilapia exhibited severe nephrotoxic alterations such as tubular vacuolation and necrosis [19,20]. Comparable lesions have also been reported in *Cirrhinus mrigala* exposed to the organophosphate monocrotophos, which induced tubular degeneration and necrosis in renal tissues [21]. These cross-species similarities demonstrate the shared patterns of renal responses to intensive farming and environmental stress.

Anatomically, fish kidneys differ from those of mammals. Unlike mammals, which possess distinct cortex and medulla zones, fish kidneys are elongated, diffuse structures located above the gas bladder and beneath the vertebral column [22]. They consist of two regions: the anterior kidney (head kidney), which functions as hematopoietic tissue with some renal tubules, and the posterior kidney (trunk kidney), which contains more renal tubules and primarily serves osmoregulatory and excretory functions [23,24,25]. Despite these structural differences, fish and mammalian kidneys share several functional similarities. One key distinction is the regenerative potential of fish kidneys [26]. In mammals, a reduced nephron number results in a permanent deficit, increasing the risk of kidney disease [27]. In contrast, teleost fish can generate new nephrons throughout life and enhance nephrogenesis following injury, thereby preserving renal function [26,28].

Immunohistochemical studies have identified fibronectin, a large adhesive glycoprotein within the extracellular matrix (ECM), as a promising biomarker for diabetic nephropathy and various glomerular disorders [29,30]. Under normal physiological conditions, fibronectin is generally localized within the glomerular basement membrane and mesangial matrix [31]. However, its expression is considerably increased in pathological states, particularly in the glomerulus and tubulointerstitial regions, where it participates in the progression of renal fibrosis [32]. Apart from its fibrotic functions, fibronectin also influences inflammatory response by supporting macrophage activation and regulating cell adhesion and migration [33]. Through interactions with transmembrane receptors, it orchestrates a range of cellular processes, including cytoskeletal organization, proliferation, differentiation, and apoptosis [34].

Recent studies have confirmed that fibronectin accumulation directly contributes to kidney dysfunction and correlates with the severity of diabetic and hypertensive nephropathies [35,36,37]. Experimental models also show that inhibition of fibronectin polymerization alleviates renal fibrosis and extracellular matrix expansion, confirming its active role in nephropathy [38,39]. These findings support its selection in the present study as a marker for evaluating renal structural remodeling in cage-farmed rainbow trout.

Renal pathologies such as diabetic nephropathy often involve a complex combination of inflammation, an inflammatory response characterized by an increased infiltration of leukocytes, regularly followed by fibrosis, and cellular stress within both the tubular and glomerular compartments, affecting various types of epithelial cells in the proximal and distal tubules [40,41,42]. Such cellular loss can result from two principal mechanisms: apoptosis and necrosis. Apoptosis is a steadily regulated physiological process essential for maintaining tissue homeostasis and eliminating damaged or potentially harmful cells [43,44]. This process is mediated by a family of cysteine–aspartic proteases known as caspases, which, once activated, initiate a cascade leading to characteristic morphological and biochemical features of programmed cell death [45,46].

Conversely, necrosis represents a passive and often uncontrolled form of cell death, typically resulting from acute cellular injury [47]. It is characterized by cellular swelling, membrane rupture, and the release of intracellular contents, which can provoke inflammation and damage surrounding tissues [41,48]. Importantly, insufficient caspase activity or impaired apoptotic signaling may lead to a shift from apoptosis to necrosis, heightening tissue injury and disease progression [49,50].

Wen et al. demonstrated that caspase-3 is important for the initiation of renal function failure, albuminuria, and tubulointerstitial fibrosis in the diabetic kidney, thereby confirming it as a key pathological executor [51]. Yang et al. established the role of caspase-3 in the pathogenesis of renal fibrosis [52]. Caspase-3 activation leads to the progressive loss of tubular and microvascular cells, which is directly linked to the development of fibrosis. Clementi et al. stated that caspases play a pivotal role in the apoptotic pathway and that caspase-3 is responsible for the progression of apoptotic mechanisms, which contributes to the advancement of renal damage [53].

The objective of this study was to investigate and compare renal tissue alterations in cage-farmed rainbow trout across different growth stages, with a particular focus on structural and molecular indicators of kidney stress. By integrating ultrastructural analysis with basic histology techniques and immunohistochemical detection of fibronectin and caspase-3 expression, we aimed to identify core morphological changes and signaling pathways involved in renal pathology. Special emphasis was placed on apoptotic processes, as a critical mechanism of cellular injury, to better understand how intensive aquaculture conditions impact renal integrity over time.

The rainbow trout (*Oncorhynchus mykiss*) is globally classified as an invasive species. The local river system (Jadro River in Solin, Croatia), which would be the only available “natural habitat” control group in this geographical area, is home to the endemic and critically protected softmouth trout, *Salmo obtusirostris salonitana*. Due to the conservation status and ethical restrictions concerning the local endemic softmouth trout, and to ensure an unbiased baseline, the normal renal histology and ultrastructure (LM and TEM) of the freshwater phase rainbow trout are defined and compared against the established literature [54,55,56,57].

Although the absence of wild or pond-farmed controls limits causal interpretation, these findings contribute to understanding renal sensitivity under aquaculture confinement and provide a foundation for future comparative and environmental studies.

## 2. Results

### 2.1. Morphological Features of the Renal Tissues in the Cage-Farmed Rainbow Trout During Different Growth Stages

**Figure 1:**
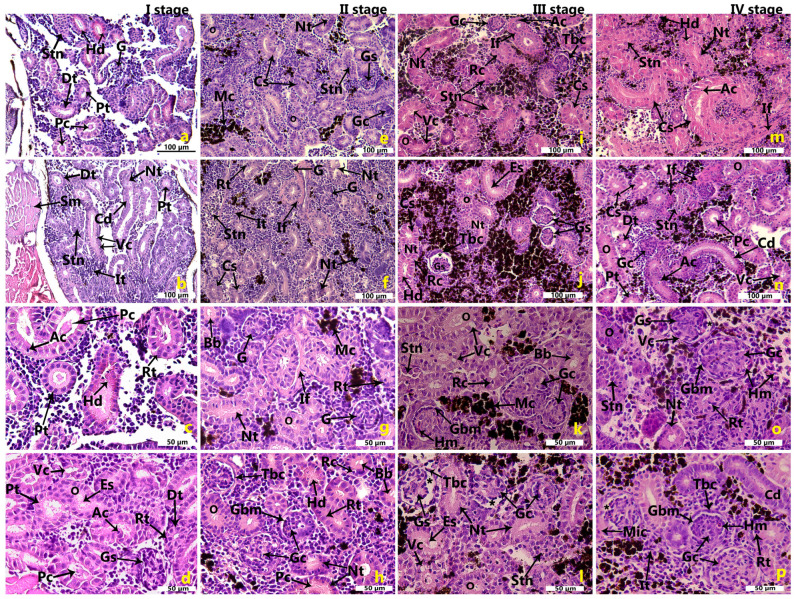
A cross-section through the kidney tissue of *Oncorhynchus mykiss* (Walbaum, 1792) during different growth stages. (**a**–**d**) Growth stage I; (**e**–**h**) growth stage II; (**i**–**l**) growth stage III; (**m**–**p**) growth stage IV. G—glomerulus; Pc—protein cast; Pt—proximal tubul; Dt—distal tubul; Cd—collecting duct; Sm—skeletal muscle; Hd—hyaline droplets; Stn—severe tubular necrosis; Nt—necrotic tubul; Vc—vacuolization; It—interstitial tissue; Ac—apoptotic cell; Rt—regenerating tubul; Es—epithelial sloughing; Gs—glomerular shrinkage; Gc—glomerular congestion; O—obstruction of luminal space; Mc—melanomacrophage centers; If—interstitial fibrosis; Bb—brush border; Hm—hemorrhage; Mic—mononuclear inflammatory cell; Rc—rodlet cell; Tbc—thickening of the Bowman’s capsule; Gbm—glomerular basement membrane; Tbm—tubular basement membrane; asterisk—widening of Bowman’s space. H&E—hematoxylin and eosin.

In tissue sections from the earliest growth stage, various cell morphologies could be identified surrounding the renal tubules. Due to cellular degeneration and loss of structural integrity, renal tubules of varying diameters were visible (Figure 1a–d). As growth stages progressed, there was a gradual increase in the number of glomeruli, renal tubules, and melanomacrophage centers, accompanied by an increase in disintegrated tubules (Figure 1e–p). Rodlet cells, a type of fish immune cell, were present and became more detectable in the later growth stages (Figure 1i,k).

One of the earliest morphological changes observed was the thickening of the tubular basement membrane, often accompanied by simplified tubular epithelial cells, which occasionally contained sloughed cellular debris, resulting in contraction of the tubular lumen (Figure 1e–p). Narrow lumens sometimes contained protein casts. Casts exhibit a distinctive appearance, often presenting with irregular, angular, and geometric shapes, and occasionally displaying a crystalline structure (Figure 1h,n). Tubular thickening, indicative of renal fibrosis, was strongly associated with tubular cell injury. As the stages advanced in growth, there was an increased degree of interstitial fibrosis and tubular atrophy. Interstitial fibrosis was characterized by excessive deposition of extracellular matrix between the tubules and peritubular capillaries (Figure 1g,m). Moreover, prominent blebbing, simplification of tubular cells, and hypertrophy of tubular cell nuclei were observed (Figure 1a–p).

Signs of tubular degeneration and necrosis were also dominant, including nuclear pyknosis, cytoplasmic degeneration, cloudy swelling, occlusion of the tubular lumen, epithelial sloughing in renal tubules, and irregular arrangement of tubular cells. This resulted in disorganized, fragmented tubules that occasionally contained vacuoles (Figure 1j,k,o). Vacuolar degeneration in tubular and glomerular cells and hydropic swelling were commonly noted. In addition to necrosis, apoptotic cells were identified as individual dark and dense fragments with condensed cytoplasm, located in the tubular epithelium and interstitial tissue. Tubules containing pyknotic nuclei indicated single-cell death, consistent with apoptosis (Figure 1a–p).

In several tubules, eosinophilic granules or hyaline droplets of varying sizes were observed in the apical part of the tubule cells across all stages. These droplets appeared as homogenous pink to dark red inclusions (Figure 1c,h,j). Severe acute tubular necrosis was characterized by the presence of masses of cells within the lumen of the tubules, with some tubules showing complete denudation of their epithelial lining and nearly total obstruction of their luminal space. Renal tubule regeneration, observed as a reparative response to prior degeneration and/or necrosis of the renal tubular epithelium, was seen across all stages. During the regeneration process following acute tubular injury, characteristic features included tubule basophilia, nuclear enlargement, and an increased number of mitotic figures. Less frequently, interstitial hemorrhages, hemorrhages within glomerular capillaries, and infiltration of mononuclear inflammatory cells were noted (Figure 1k,o,p). Early morphological changes in glomeruli included thickening of the Bowman’s capsule, often accompanied by mild glomerular congestion and hypercellularity (Figure 1h,j,l,p). This was accompanied by the disintegration of the glomerular tuft and shrinkage of glomeruli, leading to the widening of capsular spaces (Figure 1j,l).

**Figure 2:**
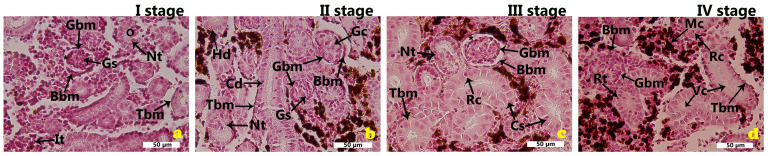
PAS staining on kidney sections of *Oncorhynchus mykiss* (Walbaum, 1792), growth stages I–IV (**a**–**d**). Cd—collecting duct; Stn—severe tubular necrosis; Nt—necrotic tubul; Vc—vacuolization, It—interstitial tissue; Gs—glomerular shrinkage; Gc—glomerular congestion; O—obstruction of luminal space; Mc—melanomacrophage centers; Bbm—Bowman’s basement membrane, Gbm—glomerular basement membrane; Tbm—tubular basement membrane; asterisk—widening of Bowman’s space.

To visualize the basement membranes of renal corpuscles and tubules, tissue sections were stained using the periodic acid-Schiff (PAS) technique. PAS staining effectively showed thickening of both glomerular and tubular basement membranes, indicating structural alterations. It also enhanced the detection of intracellular vacuoles. Rodlet cells became increasingly prominent in the third and fourth growth stages, pointing to a possible association with advancing tissue changes or immune activity (Figure 2a–d).

### 2.2. Electron Microscopy of the Renal Tissue in Cage-Farmed Rainbow Trout

**Figure 3:**
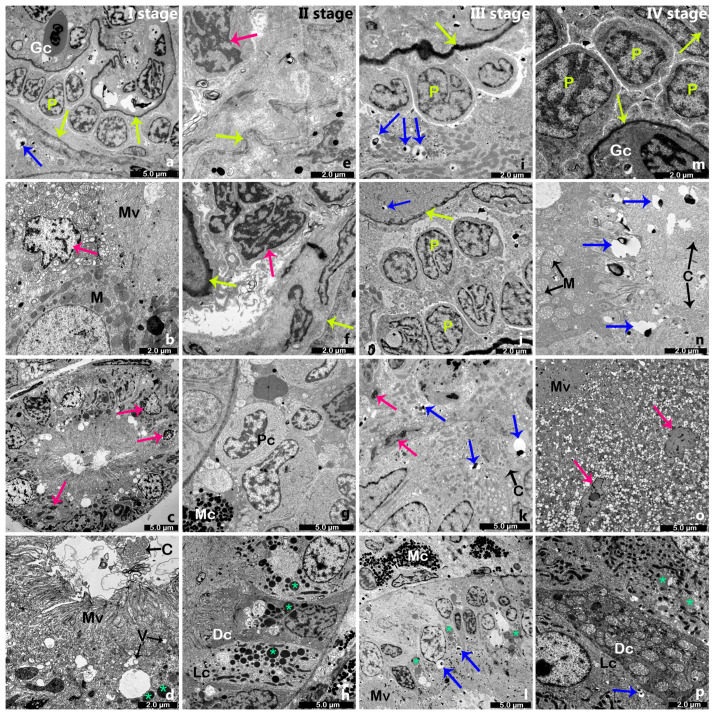
A cross-section throughout the kidney tissue in four growth stages of rainbow trout, *Oncorhynchus mykiss* (Walbaum, 1792). (**a**–**d**)—growth stage I; (**e**–**h**)—growth stage II; (**i**–**l**)—growth stage III; (**m**–**p**)—growth stage IV. Pink arrows—apoptotic cells and nuclei; blue arrows—apoptotic bodies; yellow arrows—capsular and glomerular basement membranes; Gc—glomerular capillary; P—podocytes; M—mitochondria; C—cilia; Mc—melanomacrophage centers; Pc—plasma-like cell; Mv—microvilli; V—vesicle; L—light cell; Dc—dark cell; asterisk—lipid droplets. Magnification—((**a**,**l**,**o**) ×1200; (**b**,**e**,**j**,**p**) ×2500; (**c**) ×800; (**d**,**f**,**i**,**n**) ×3000; (**g**,**h**,**k**) ×1500; (**m**) ×4000), TEM.

Stage I. Ultrastructural analysis of kidney tissues from the first growth stage revealed podocytes lying along the capillary basement membrane, extending their foot processes over the capillaries. The podocytes are pale with poor cytoplasm and large almost euchromatic nucleus (Figure 3a). Epithelial cells lining tubules were abundant in cytoplasm, with distinct mitochondrial clusters located in the apical (proximal tubule) and basal regions (distal tubule) of the cells (Figure 3b–d). Numerous decaying mitochondria, characterized by degraded and hollow cristae, were observed. In the apical part of the proximal tubule, long-protruded microvilli were seen (Figure 3b). Occasional tubules showed shrunken, probably apoptotic cells with condensed cytoplasm alongside irregularly shaped nuclei (Figure 3c). Granules, corresponding to eosinophilic ones observed under H&E staining, were identified in certain apical regions of the tubular cells. In sections encompassing the entire proximal tubule, the presence of cilia and microvilli was noted. Microvilli projected from the apical portion of the cells, while cilia exhibited a regular arrangement, with cross sections revealing the characteristic 9 + 2 microtubular structure (Figure 3b). Certain tubular regions contained numerous lipid droplets, along with melanomacrophage centers (Figure 3d).

Stage II. Several ultrastructural changes could be observed as growth progressed. During stage II, the basement membrane of Bowman’s capsule as well as the glomerular basement membrane appeared irregular and more thickened (Figure 3e,f). In some glomeruli, mesangial cells with irregularly shaped nuclei were described (Figure 3f). The presence of both cilia and microvilli was observed. Continuous presence of cilia appears to be associated with tubular injury and interstitial fibrosis (Figure 3d,k,n). Within the interstitium, apart from numerous melanomacrophage centers, a variety of inflammatory cells were also present, including plasma-like cells and leukocytes (Figure 3g). The cytoplasm contains rough endoplasmic reticulum, vacuoles of varying sizes, and free ribosomes. The epithelium of several tubules forms two distinct cell types: light cells and dark cells, both filled with numerous lipid droplets (Figure 3h,p). In the basal region of the distal tubule, invaginations of the basement membrane were evident, along with elongated mitochondria.

Stage III. Within the renal corpuscle of the kidney tissue from the third growth stage, numerous apoptotic bodies surrounding podocytes were observed (Figure 3i). Closely aligned podocytes with pale cytoplasm and euchromatic nuclei could be seen communicating with each other by foot processes (Figure 3j). Apoptotic bodies, as well as apoptotic degenerative nuclei with chromatin condensation, were observed even in the epithelial cells of tubules (Figure 3k). In the proximal tubule, numerous melanomacrophage centers and lipid droplets were observed (Figure 3l) along with several dark, condensed, and often elongated nuclei, indicating whether necrosis or apoptosis was present. Vacuoles and vesicular bodies were identified in the apical region of the proximal tubule. These findings further underscore the extent of renal damage at this stage.

Stage IV. Podocytes within renal corpuscles from stage IV kidneys differed in their ultrastructure in comparison to those from the three previous stages. Their cytoplasm is more abundant, and the nucleus is heterochromatic (Figure 3m). Epithelial cells of the proximal tubules displayed cilia with basal bodies clearly visible. Necrotic morphological changes were evident, including enlarged and swollen mitochondria undergoing disintegration, loss of matrix integrity, and the presence of numerous apoptotic bodies (Figure 3n). Whether necrotic or apoptotic cells with distorted, pyknotic nuclei were also identified (Figure 3o). In the proximal tubule epithelium, the cellular shrinkage and membrane blebbing, as well as nuclear fragments of varying sizes and shapes composed of condensed chromatin, were observed. Both light and dark cells were also noted. Light cells, which are cuboidal in shape, lacked microvilli but contained irregularly shaped mitochondria, rough endoplasmic reticulum, and free ribosomes. In contrast, dark cells exhibited electron-dense cytoplasm filled with numerous mitochondria and an abundance of smooth endoplasmic reticulum. Their apical membranes contained long microvilli (Figure 3h,p).

### 2.3. Expression of Fibronectin and Caspase-3

**Figure 4:**
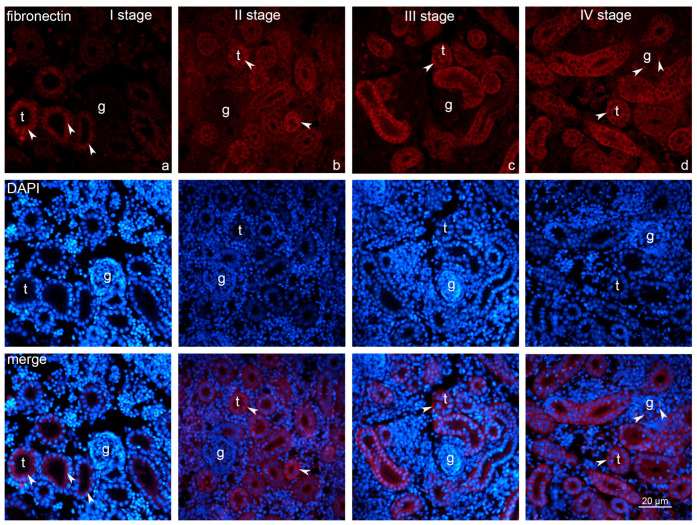
Expression of fibronectin in the renal tissues of the *Oncorhynchus mykiss* (Walbaum, 1792) within four different growth stages, stage I–IV (**a**–**d**): g—glomerulus; t—renal tubules; arrowheads—positive labeling of fibronectin. Magnification—40×, scale bar 20 μm.

**Figure 5:**
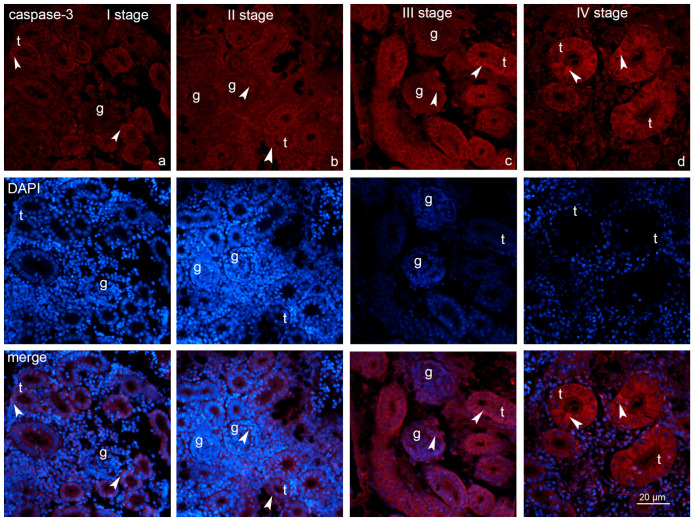
Expression of caspase-3 in the renal tissues of the *Oncorhynchus mykiss* (Walbaum, 1792) within four different growth stages, stage I–IV (**a**–**d**): g—glomerulus; t—renal tubules; arrowheads—positive labeling of caspase-3. Magnification—40×, scale bar 20 μm.

Immunofluorescence microscopy uncovered the apparent progressive intensification of fibronectin expression throughout the growth stages. Positive labeling for fibronectin was predominantly localized within the tubules across all growth stages (Figure 4). Comparatively, glomerular expression was absent during stages I, II, and III, but weak labeling was detected in stage IV. Caspase-3-positive cells were present in the renal tubules of all growth stages. In comparison with glomerular expression that was absent during stage I but became detectable from stage II onward, persisting through stages III and IV (Figure 5). Immunofluorescence microscopy of kidney sections from rainbow trout demonstrated that fibronectin and caspase-3 immunoreactivity were predominantly localized to distinct subcellular regions within both proximal and distal tubular epithelial cells, with occasional positive staining also observed in glomerular cells.

### 2.4. Statistical Analysis of the Fibronectin and Caspase-3 Measurements

**Figure 6:**
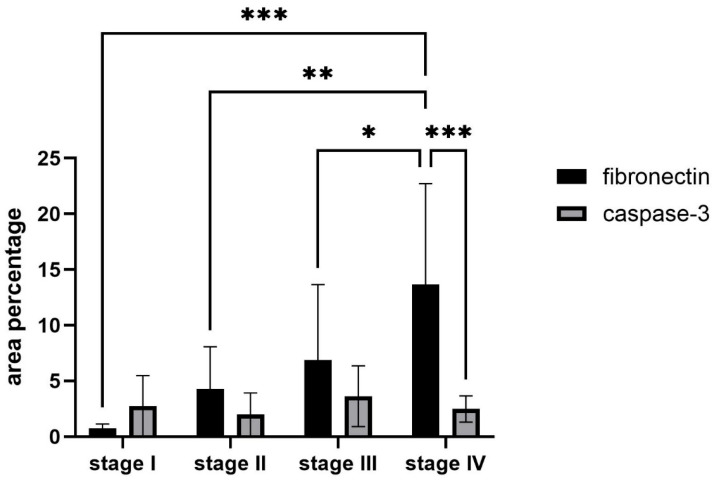
Statistical analysis of the expression pattern of fibronectin and caspase-3 in rainbow trout kidney across four growth stages. Expression was quantified by measuring the percentage of immunoreactive cell areas (% area). Fibronectin expression increased significantly at stage IV compared with stages I (*** *p* < 0.001), II (** *p* < 0.01), and III (* *p* < 0.05). Caspase-3 expression showed no significant differences among stages. At stage IV, fibronectin expression was significantly higher than caspase-3 (*p* < 0.001). Data were analyzed and confirmed with two-way ANOVA, Šídák’s, and Tukey’s multiple comparisons tests.

Quantitative analysis of renal samples across four growth stages of rainbow trout showed marked differences in fibronectin expression, while caspase-3 levels remained relatively stable. It was confirmed that fibronectin expression increased progressively with advancing stages, with the most pronounced elevation detected at stage IV. Fibronectin levels in stage IV were significantly higher compared to stage I (Figure 6, *p* < 0.001), stage II (Figure 6, *p* < 0.01), and stage III (Figure 6, *p* < 0.05). This pattern suggests that fibronectin deposition becomes increasingly prominent in later stages, possibly due to progressed morphological changes.

In contrast, caspase-3 expression showed no statistically significant variation across the four stages. This indicates that apoptotic activity, as measured by caspase-3, remained relatively constant during kidney maturation and did not follow the same stage-dependent escalation observed for fibronectin. However, a significant difference was observed in stage IV when directly comparing fibronectin and caspase-3 expression (Figure 6, *p* < 0.001), showing a difference between extracellular matrix accumulation and apoptosis regulation at this advanced stage.

### 2.5. Semi-Quantitative Analysis of the Morphological Outcomes of the Rainbow Trout Kidney

#### 2.5.1. Semi-Quantitative Analysis of the Morphological Outcomes of the Rainbow Trout Kidney Within Each Growth Stage

**Figure 7:**
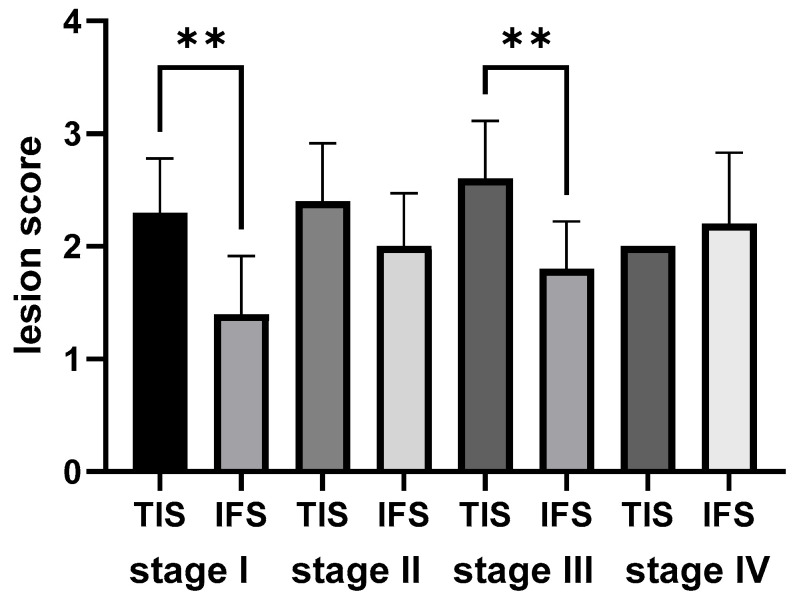
Comparative statistical analysis of the morphological changes in the rainbow trout kidney within each growth stage. Morphological progression was assessed using a semi-quantitative scoring system for both tubular injury and interstitial fibrosis. Data were analyzed with the Kruskal–Wallis test and Dunn’s test for Post Hoc analysis. ** *p* < 0.01; TIS—tubular injury score; IFS—interstitial fibrosis score.

Histopathological examination revealed that tubular injury and interstitial fibrosis were present in the kidneys of trout across all four growth stages. Subsequent comparative analysis was performed to evaluate the dynamic progression of these morphological changes. The results provided insight into the comparative severity of tubular injury (TIS) and fibrosis (IFS) within different growth stages. In the fry stage, stage I, TIS scores were significantly higher than IFS scores (*p* < 0.05), indicating that tubular injuries were more pronounced than fibrosis. In contrast, no significant difference was observed between TIS and IFS scores in stage II (*p* > 0.05), suggesting both changes were comparably expressed. The pattern repeated in the later stages; TIS scores were significantly higher than IFS scores in stage III (*p* < 0.05), while no significant difference was found in stage IV (*p* > 0.05). The observed pattern indicates that the dominance of tubular injury over fibrosis is not constant during growth. TIS prevails over IFS in some phases (stages I and III), but their severity levels equalize in others (stages II and IV) (Figure 7).

#### 2.5.2. Semi-Quantitative Analysis of the Morphological Outcomes of the Rainbow Trout Kidney Across Four Growth Stages

**Figure 8:**
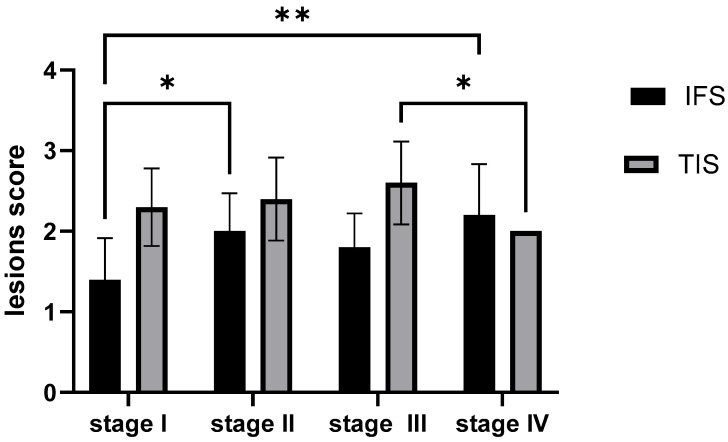
Comparative statistical analysis of the morphological changes in the rainbow trout kidney across four growth stages. Histopathological progression was assessed using a semi-quantitative scoring system for both tubular injury and interstitial fibrosis. Data were analyzed with two-way ANOVA and Tukey’s multiple comparisons test. ** *p* < 0.01; * *p* < 0.05; TIS—tubular injury score; IFS—interstitial fibrosis score.

A significant change in tubular injury is observed only between stages III and IV (*p* = 0.0323; *p* < 0.05), where the score increases. In earlier stages and between earlier and later stages, there is no significant difference. This could indicate that tubular injury significantly worsens only in the latest stage. A highly statistically significant difference in the mean fibrosis score is found between stage I and stage II (*p* = 0.0323; *p* < 0.05) as well as between stage I and stage IV (*p* = 0.0021; *p* < 0.01). Fibrosis significantly increases between the earlier stage I and the later stages II and IV, but there are no significant changes between the later stages themselves (Figure 8).

Taken together, the comparison of TIS and IFS, first within each stage and then across all stages, shows that in earlier phases (stage I), acute tubular injury is dominant, and fibrosis is just developing. Over time, fibrosis progresses (significantly from stage I to II and IV), while acute tubular injury significantly worsens only in the last stage IV. The observed pattern indicates that the dominance of tubular injury over fibrosis is not constant during growth. TIS prevails over IFS in some phases (stages I and III), but their severity levels equalize in others (stages II and IV). This could suggest that in earlier phases, tubular injury triggers fibrosis development, and in later phases, chronic fibrosis and acute tubular injury can worsen concurrently, which is a typical scenario in the progression of kidney disease.

## 3. Discussion

The histological and ultrastructural analyses revealed progressive renal alterations in cage-farmed rainbow trout, predominantly affecting the tubular epithelium and glomerular architecture. Degenerative changes such as vacuolation, epithelial sloughing, and mitochondrial disruption were evident in all growth stages, supporting the interpretation that prolonged confinement and rearing practices impose chronic stress on renal tissues [6,58,59,60]. Similar renal lesions have been described in *Cyprinus carpio* exposed to deltamethrin, showing pronounced glomerular and tubular degeneration [17], and in *Oreochromis niloticus* reared under chronic ammonia exposure, where tubular necrosis and congestion were also reported [18]. Comparable nephrotoxic effects have been observed in *Clarias gariepinus* exposed to organophosphate insecticides such as Sniper 1000EC, which induced tubular degeneration, nephrosis, and epithelial hyperplasia [5], and in *Ophiocephalus striatus* subjected to pyrethroid exposure, resulting in glomerular shrinkage, tubular necrosis, and nuclear abnormalities [60]. Collectively, these findings reveal a conserved pattern of renal response among teleost species facing chemical, environmental, or culture-related stressors.

Burlaka et al. (2016) described that in proteinuric kidney disease, excessive albumin filtration induces apoptosis in both podocytes and tubular epithelial cells, leading to glomerular-tubular disconnection and progressive renal dysfunction [61]. Therapeutic intervention with ouabain, which modulates the Na,K-ATPase/IP3R signaling pathway, can prevent apoptosis, preserve renal structure, and maintain kidney function [61]. This shows the potential of targeting apoptotic pathways to reduce renal injury. However, some studies reported that prolonged antibiotic exposure caused tubular epithelial disruption, necrosis, vacuolation, and glomerulopathy, suggesting that even therapeutic agents can complicate renal health when misused or overapplied [19,20,62]. Together, these studies demonstrate the sensitivity of renal tissues to a wide group of stressors [63,64]. The accumulation of cellular debris, inflammatory infiltrates, and fibrotic changes observed across these models supports the understanding that the renal tubules are often primary targets and active participants in the progression of kidney pathology [65,66].

The simultaneous presence of both cilia and microvilli observed in our study may reflect a pathological response in the tubular epithelium [67]. In Rabah’s study, acute Taxol exposure led to visible destruction of the proximal tubular brush border, involving the loss and disorganization of apical microvilli [68]. Primary cilia are known to serve as mechanosensory and chemosensory organelles that regulate key signaling pathways involved in epithelial cell differentiation, polarity, and repair [69]. Some studies reported that disruption in ciliary structure or function is related to tubular injury and interstitial fibrosis, as seen in renal ciliopathies such as cystic kidney disease and nephronophthisis [70]. The altered expression or persistence of cilia in injured tubules may indicate disrupted repair processes or sustained epithelial stress [71]. Therefore, our findings may point to early signs of defective repair and the value of ciliary signaling in maintaining tubular structure.

Apoptotic cell changes and apoptotic bodies were detected from the earliest stage examined (stage I). Similar early changes have also been described in zebrafish [72] and goldfish [73], showing that apoptosis can begin early in fish development under stress. Glomerular damage was characterized by congestion, shrinkage, and frequent thickening or disruption of Bowman’s basement membrane. Podocytes were often observed among apoptotic debris, suggesting their involvement in the modification of glomerular cells [74]. Tubular changes included epithelial sloughing, narrowing of the tubular lumen, and the accumulation of proteinaceous casts as indicators of acute tubular injury. These casts were identified based on their characteristic appearance as homogeneous, eosinophilic intraluminal material lacking nuclei, consistent with descriptions of proteinaceous casts in vertebrate renal pathology [75]. At the ultrastructural level, affected cells exhibited swollen, disfigured mitochondria with distorted cristae, irregular and apoptotic nuclei, vesicle accumulation, lipid droplets, and inflammatory infiltrates. Fibrotic changes were also noted, particularly in the form of dense extracellular matrix deposits between tubules, indicating the start of interstitial scarring [76,77].

Dynamic changes in rainbow trout kidney tissues across different growth stages reflect a well-known pathological principle in kidneys, not only in fish but also in mammals: renal tubular injury often precedes or is closely associated with the development of interstitial fibrosis. In cases of chronic or recurrent tubular injury, epithelial cells can undergo maladaptive repair [78], leading to an inflammatory response and the activation of fibroblasts [79]. This results in the accumulation of extracellular matrix and fibrosis [80,81]. In our study, the imbalance observed in stages I and III could indicate periods of acute injury, where tubular damage is the primary event. Conversely, the balance between TIS and IFS in stages II and IV might reflect a more chronic phase, in which the fibrotic response has caught up with the initial tubular damage, or it could indicate periods of partial repair and regeneration. Fibrosis is often a chronic response to persistent injury. In the acute phases, the injury is dominant, while the fibrotic response is nascent. Additionally, it is possible that, in stages I and III, a new cycle of injury or an increased effect of a harmful factor occurred. This dynamic interplay underscores the complexity of progressive kidney tissue changes in rainbow trout and suggests that the response to chronic or repeated insults is not linear.

Localized interstitial fibrosis was observed around degenerated tubules, marked by extracellular matrix expansion and fibronectin accumulation. These focal lesions indicate partial scarring of the renal interstitium, consistent with chronic reparative responses to sustained stress [39,65,66,76,77,82]. Similar limited fibrotic patterns have been reported in other teleosts under chronic pollutant or crowding stress [58,59]. The fluctuations in the ratio of tubular injury to interstitial fibrosis likely reflect cycles of acute injury, chronic response, and regeneration. These findings suggest a dynamic shift in kidney pathology, with initial stages characterized by dominant acute tubular injury (stage I), which may trigger progressive fibrotic changes observed from stage I onwards. Later stages, particularly stage IV, show a significant worsening of tubular injury alongside established fibrosis, indicating a complex interplay between these changes as growth progresses.

It was interesting to note that alongside evidence of apoptosis and necrosis, regenerative activity was observed in the form of mitotically active, basophilic cells [83,84]. This suggests that kidney tissue was attempting to repair and replace damaged cells, indicating there is a balance between cell death and restoration that is critical for maintaining organ function [85]. Apoptosis, a regulated form of programmed cell death, plays a fundamental role in this process [43,86]. It involves chromatin condensation, DNA fragmentation, and the formation of apoptotic bodies, which are membrane-bound vesicles containing cellular components [48]. These are rapidly cleared by phagocytes, allowing for the non-inflammatory removal of damaged cells [87]. In addition to this clean-up function, apoptotic bodies may also serve a signaling role by delivering bioactive molecules to neighboring cells, influencing immune responses, and potentially supporting tissue regeneration [88].

Distinguishing apoptosis from necrosis can be challenging using conventional histological techniques, especially when both occur at the same time [48]. The nature of the stimulus, energy availability, and the presence of active caspases all influence which form of cell death predominates [89]. Among the caspase family, caspase-3 is a central executioner enzyme in apoptosis [46]. Once activated through intrinsic or extrinsic pathways, it cleaves many structural and regulatory proteins, ultimately triggering the structural breakage of the cell [90,91]. Due to its vital role, caspase-3 is generally used as a molecular marker to detect and quantify apoptosis [46].

Fibronectin’s dual role as a structural extracellular matrix (ECM) glycoprotein and as a mediator of fibrosis has been well established in both mammalian and teleost kidneys [29,30,35,36,39,76,77]. In mammalian nephropathies, elevated fibronectin expression accompanies glomerular and tubular injury, and its inhibition reduces fibrotic progression, confirming its active involvement in disease development [29,30,39,76,77]. In the present study, its spatial localization around degenerated tubules and interstitial fibrotic areas supports a pathological rather than developmental interpretation, indicating that fibronectin accumulation reflects stress-associated remodeling rather than normal growth.

At the same time, fibronectin’s biological versatility should be acknowledged. While its overexpression is often linked to fibrotic progression, it also exhibits cytoprotective and anti-apoptotic functions that can promote early tissue repair [92,93,94]. This dual functionality suggests that fibronectin contributes to both short-term preservation of tissue integrity and to long-term matrix expansion under chronic stress [95,96,97,98]. In acute injury, its anti-apoptotic properties may aid recovery, whereas during prolonged confinement and metabolic stress, such as those characteristics of cage aquaculture, continued expression may amplify inflammatory and fibrotic signaling [33,39]. Thus, fibronectin’s increased intensity across growth stages in rainbow trout represents a complex response, integrating both reparative and probably pathological processes associated with sustained environmental stress.

Our data show that fibronectin expression in the rainbow trout kidney is not merely a function of organismal growth. Instead, its increase, especially in stage IV, seems to be driven by the progressive morphological stress associated with cage farming. Fibronectin is known as a matrix protein involved in repair and fibrosis, and its expression in stage IV, when tissue differentiation is complete, suggests a response to chronic stress or injury rather than developmental maturation [99]. Caspase-3, in contrast, showed a pattern consistent with its role in programmed cell death. Higher expression in stage I agrees with reports in zebrafish and goldfish showing that caspase-3 activity peaks during early development, when apoptosis contributes to organogenesis [72,73]. As the kidney matures, caspase-3 expression oscillates, indicating the reduced requirement for developmental apoptosis. The persistence of caspase-3 signals at later growth stages is likely attributable to stress-related apoptosis induced by intensive farming conditions, including limited space and exposure to treatments, rather than to growth processes. High variability in caspase-3 activity in the renal tubules and glomeruli during development likely reflects its diverse, context-specific functions beyond simple cell death. The continuous presence of Caspase-3 positive cells in the tubules and its distinct, delayed appearance in the glomeruli suggest that different enzyme activity forms are responsible for specific developmental and maintenance processes in each compartment [100].

The presence of inflammatory cells further affirmed the ongoing injury and repair processes. Fish begin life as independent organisms from their earliest age and rely heavily on innate immune responses to ensure survival [101]. This nonspecific immune system serves as their primary line of defense against pathogens [102]. Among the most found white blood cells in their hematopoietic tissues are lymphocytes, neutrophils, and macrophages [103]. Tissue damage initiates an immune response marked by leukocyte infiltration [104]. Ghielli et al. reported that such cells persist at injury sites long enough to support regeneration, after which they undergo apoptosis [105]. However, if not efficiently regulated, leukocytes can complicate injury by releasing reactive oxygen species and proteolytic enzymes, promoting further tissue damage and fibrotic changes [106]. Studies on fish exposed to environmental contaminants or immunological stressors have shown high recruitment of neutrophils and macrophages to damaged tissues, where they can either support repair or contribute to morphological changes, depending on the intensity and regulation of the response [107].

Backing these observations, a recent study by Liu et al. demonstrated that prolonged exposure to mequindox (MEQ) in mice induced marked mitochondrial damage and apoptosis in renal tissues [50]. Transmission electron microscopy revealed mitochondrial vacuolation and cellular degeneration in both cortex and medulla, accompanied by nuclear shrinkage and apoptotic body formation. MEQ exposure resulted in activation of caspases 3, 8, and 9. These effects happened together with strong oxidative stress, showing that both cell death and inflammation act as warning signs of kidney damage caused by toxins in line with findings in fish [108] and humans with chronic kidney disease [109]. These findings point out that persistent inflammation in the renal interstitium may be a crucial factor in the progression of tubular injury, bringing attention to the importance of maintaining immune balance to prevent the transition from possible repair to chronic pathology.

In our study, the detection of leukocytes and plasma-like cells, particularly in the tubulointerstitial region, suggests ongoing inflammation. Numerous studies have confirmed that the localization of immune cells within the interstitium during acute kidney injury is strongly associated with tubular damage and may even contribute to regeneration [33,105,110]. Supporting this, we observed cytological alterations such as tubular basement membrane thickening and signs of both tubular atrophy and regeneration. Additionally, hyaline droplets, vacuolation, and fibroblastic responses in the interstitial space indicated progressive tissue alteration [111,112]. Immune cells such as plasma-like cells, responsible for long-term antibody production in teleosts, and rodlet cells, known for their defensive role against parasites, were also detected [113,114]. Rodlet cells are typically localized within epithelial tissues of mucosal organs such as the gills, intestine, and skin, where they participate in immune and secretory functions [115]. However, their occasional occurrence within the renal interstitium has been documented in stressed or diseased fish and may reflect a systemic immune response or stress-induced recruitment of rodlet cells from deeper tissues [114]. The presence of the rodlet cells within the kidney, specifically in the tubular epithelium, Bowman’s capsule, or interstitial hematopoietic tissue, is a well-documented histological indicator of chronic stress, disease, or systemic immune response even in salmonids [116,117].

The appearance of melanomacrophage centers, which serve as depositories for cellular debris and degraded erythrocytes, further confirmed an ongoing immune and clearance response [118]. These structures increased in number and size with fish age and tissue degeneration, in line with our observations across growth stages.

Although glomerular alterations were apparent, many morphological changes were localized to the renal tubules. This observation aligns with recent shifts in nephrology research [119]. While glomerular damage has generally been viewed as the primary driver of chronic kidney disease (CKD) [120,121], there is now a greater focus on the vulnerability of tubular epithelial cells [122]. Due to their high metabolic demand, tubular cells are particularly susceptible to ischemia, toxins, and oxidative stress [110,123,124]. Tubular injury is now recognized as a major contributor to interstitial inflammation and fibrosis, which are critical predictors of CKD progression [125]. Our findings, characterized by increasing expression of nephropathic markers and structural deterioration in tubular regions, strongly validate this focus of change. The central role of tubular health in renal pathology implies that both diagnostic and therapeutic strategies should address not only glomerular function but also the integrity of the tubulointerstitial compartment [66].

Despite these consistent observations, several limitations must be acknowledged. The absence of a control group from a natural or semi-natural habitat prevents definitive attribution of renal damage solely to cage-farming conditions. Due to the conservation status and ethical restrictions concerning the local endemic softmouth trout (*Salmo obtusirostris salonitana*), and to ensure an unbiased baseline, the normal renal histology and ultrastructure (LM and TEM) of the freshwater phase *Oncorhynchus mykiss* are defined and compared against the established literature [54,55,56,57]. This allows for precise documentation of progressed morphological changes observed within the intensively farmed cohort.

## 4. Materials and Methods

### 4.1. Collection of Samples

The fish were collected from a fish farm located in Solin, Croatia, where they were held in raceways 1.1 m deep with consistent water exchange rates every 20 min, a water source from the spring of the river Jadro, and excellent quality parameters (water temperature 18 °C, pH from 7 to 8, dissolved O_2_ ≥ 8 mg/L, near 100% saturation, ammonia < 0.02 mg/L, nitrite < 0.1 mg/L, CO_2_ < 10 mg/L, moderate alkalinity). The fish density was 5–50 trout/m^2^ (in early growth) and up to 20 kg/m^2^ under good conditions in the raceway. Fish health monitoring was conducted twice daily for early detection and routine observations. Diagnostic sampling was performed by weekly checks on random fish for external parasites, gill health, and bacterial cultures. Prophylactic treatment followed veterinary regulations.

The study was conducted on 40 rainbow trout *Oncorhynchus mykiss* (Walbaum, 1792) specimens in February 2023, representing four distinct growth stages, with ten specimens analyzed for each stage. Fish were classified into four age groups: the youngest group representing the fry stage, followed by two middle groups representing the juvenile stage, and concluding with the last group representing the reproductively matured (adult) stage (Table 1). After being freshly caught, the specimens were immediately transported on ice to ensure their preservation. Upon arrival at the Laboratory for Histology and Electron Microscopy at the Department of Biology, Faculty of Science, University of Split, the length and weight of each specimen were measured. Kidney tissues were then carefully extracted from all samples and stored according to the specific requirements of the research techniques to be performed.

### 4.2. Renal Tissue Preparation

For basic histology, histochemistry, and immunofluorescence, the tissues were preserved in 4% paraformaldehyde. For transmission electron microscopy, the tissues were stored in McDowell fixative. The fixative solution was prepared by combining 40 mL of 25% glutaraldehyde, 100 mL of 36% formaldehyde, 860 mL of distilled water, 2.7 g of NaOH, and 11.6 g of NaH_2_PO_4_ × 2H_2_O, adjusted to a final volume of 1 L. Tissue sections prepared for histology, histochemistry, and immunofluorescence were embedded in paraffin blocks and sectioned using a rotary microtome HistoCore BIOCUT (Leica, Wetzlar, Germany). For electron microscopy, the samples were embedded in resin and sectioned with an ultramicrotome (PowerTome XL, RMC Boeckeler, Boeckeler Instruments, Inc., Tucson, AZ, USA). All techniques were conducted following their protocols, respectively.

#### 4.2.1. Hematoxylin–Eosin (H&E)

Tissue samples were cleared with xylene and embedded in paraffin blocks. The deparaffinized tissue sections mounted on glass slides were stained using hematoxylin–eosin. Hematoxylin was used to stain the nuclei a dark purple, while eosin served as a counterstain, coloring the cytoplasm and other structures, including the extracellular matrix, in various shades of red.

#### 4.2.2. Periodic Acid Schiff—PAS

Deparaffinized and rehydrated tissue sections were treated with 0.5% periodic acid solution, rinsed in distilled water, and immersed in Schiff reagent. The sections were then washed in tap water, counterstained with Mayer’s hematoxylin, rinsed again in lukewarm tap water, dehydrated, and finally mounted with a synthetic mounting medium under a coverslip. Microscopic slide examination was conducted using a light microscope, Leica DM 3000, LED (Leica, Wetzlar, Germany) equipped with Leica DMC 4500 (Leica, Wetzlar, Germany) camera.

### 4.3. Immunofluorescence

Tissue sections were first deparaffinized with xylene and rehydrated through a graded ethanol series. Subsequently, the sections were heated in citrate buffer (pH 6.0) for 8–10 min, a step necessary to unfold antigen epitopes for primary antibody binding. The sections were then allowed to cool at room temperature for 20 min. After rinsing in Phosphate-Buffered Saline (PBS, pH 7.2), the slides were placed in a humid chamber. Once dried, a hydrophobic pen (PAP pen) was used to encircle the tissue sections to prevent leakage of solutions during subsequent steps. A few drops of protein block solution were applied to each section to block nonspecific binding of the primary antibodies. Diluted primary antibody was then applied to the tissue sections in carefully calculated proportions. The primary antibodies used were mouse monoclonal Anti-Fibronectin antibody (F7387, Sigma-Aldrich Co., St. Louis, MO, USA) and rabbit polyclonal anti- Caspase-3 antibody (SAB5701085, Sigma-Aldrich Co., St. Louis, MO, USA (UniProt acc. no. P42574)), both diluted at 1:200. The slides were incubated overnight. The following day, the sections were rinsed in PBS, and secondary antibodies diluted in PBS were applied and incubated for one hour in the dark. The secondary antibodies used were goat Anti-Mouse IgG (Alexa Fluor 647) (ab150115; Abcam, Cambridge, UK) and donkey polyclonal Anti-Rabbit IgG (Alexa Fluor 488) (711-545-152, Jackson Immuno Research Laboratories, Inc., Baltimore, PA, USA), both diluted at 1:400. After rinsing with PBS, DAPI dye (4′,6-Diamidino-2-Phenylindole Dihydrochloride) was used to stain nuclei. The sections underwent a final PBS rinse before being mounted (Aqua-Poly/Mount, Polysciences Inc., Warrington, PA, USA) and covered with a coverslip. Microscopic examination was performed using an Olympus BX51 (Tokyo, Japan) epifluorescence microscope with a Nikon DS-Ri1 digital camera (Nikon Corporation, Tokyo, Japan). The negative controls were done following the same procedure but without applying the primary antibody.

### 4.4. Transmission Electron Microscopy

Tissue sections were fixed in the McDowell fixative. After fixation, the sections were washed in phosphate buffer, followed by postfixation in 2% osmium tetroxide (OsO_4_). The specimens were then washed again in phosphate buffer, dehydrated through a graded ethanol series, and subsequently infiltrated and embedded in Spurr resin. Semi-thin sections were cut at 500 nm, and the area of interest was chosen for ultra-thin sectioning. Ultra-thin sections were cut at 60 nm using Diatome diamond knives (Diatome Ltd., Nidau, Canton Bern, Switzerland) and placed onto copper grids. The sections were then stained with uranyl acetate and lead citrate for contrast enhancement and examined under a transmission electron microscope, JEM JEOL 1400 Flash (JEOL, Tokyo, Japan).

### 4.5. Statistical Analysis

#### 4.5.1. Quantitative Analysis of the Fibronectin and Caspase-3 Expression

Fibronectin and caspase-3 expression in kidney tissue of rainbow trout was quantified using ImageJ software 1.8.0 (National Institutes of Health, Bethesda, MD, USA). For each sample, twenty non-overlapping representative visual fields were analyzed at 40× magnification under identical exposure conditions. The percentage of fibronectin and caspase-3 immunoreactive areas and fluorescence intensity were measured. For immunoreactivity analysis, photomicrographs were processed by subtracting the median filter and applying color thresholding to determine the percentage area occupied by positive signals [126,127].

For statistical analysis, the area percentage and expression levels of fibronectin and caspase-3 were statistically analyzed using two-way ANOVA, followed by Tukey’s multiple comparisons test. Results are presented as mean ± standard deviation (M ± SD). Statistical significance was set at *p* < 0.05. All analyses were performed using GraphPad Prism version 10.1.1 (GraphPad Software, Inc., San Diego, CA, USA).

#### 4.5.2. Semi-Quantitative Analysis of the Morphological Outcomes (Tubular Injury and Fibrosis)

To fully address the morphological parameters (tubular degeneration, atrophy, necrosis, and interstitial fibrosis), which are typically assessed across larger fields of view and often require specific staining (H&E), we performed a semi-quantitative scoring system. A semi-quantitative scoring system for tubular injury and interstitial fibrosis was used to assess the morphological progression of damage systematically. This method relies on visual estimation of the percentage of affected tissue area, a standard procedure in renal pathology and ecotoxicological studies. Our specific scoring criteria (0–4) were developed based on systems for grading tubular damage and interstitial fibrosis per field of view (0 is normal; 1 is <25%; 2 is 25% to 50%; 3 is 51% to 75%; and 4 is >75%) [128,129,130,131]. This approach provides a robust, semi-quantitative measure of pathology, which complements our fully quantitative analyses of fibronectin and caspase-3 areas.

Descriptive statistics, including medians and frequencies, were calculated for the pathology scores in each growth stage. To test statistically significant differences in pathology scores among the four growth stages, two-way ANOVA and Tukey’s multiple comparisons test were used. The Kruskal–Wallis test and Dunn’s test for post hoc analysis were used to assess statistically significant differences in pathology scores within each growth stage. The significance level of *p* < 0.05 was considered statistically significant. The analysis was conducted using GraphPad Prism version 10.1.1 (GraphPad Software, Inc., CA, USA).

## 5. Conclusions

This study confirms significant morphological changes in kidney tissues in cage-farmed rainbow trout, with recognizable effects on glomeruli and especially severe alterations in the renal tubules exposed to environmental and chemical stressors in aquaculture settings. Histological and ultrastructural alterations observed across different growth stages, including signs of apoptosis, necrosis, inflammation, and fibrosis, reflect an interaction between injury and repair mechanisms. Markers such as fibronectin and caspase-3 revealed ongoing cellular modifications. Our results align with the increasing number of studies that feature the renal tubules as critical mediators of kidney injury and disease progression. The detection of inflammatory cells, apoptotic bodies, and degenerative changes as early as the fry stage clarifies that environmental confinement, nutritional practices, and the use of prophylactic treatments may collectively contribute to the development of nephropathy and have cumulative and long-lasting impacts on fish health. These findings urge the adoption of more acceptable aquaculture practices, careful monitoring of chemical use, and a broader understanding of renal function beyond glomerular damage to ensure the welfare and productivity of farmed fish. Further research should be conducted with additional attention to the expression patterns of molecular markers to deepen our understanding of renal injury progression and regulation under intensive farming conditions.

## Figures and Tables

**Table 1:** Classification of the *Oncorhynchus mykiss* (Walbaum, 1792) specimens.

	Stage I	Stage II	Stage III	Stage IV
Age (months)	1	11	13	18
Avg. length (cm)	3.885	15.33	21.68	27.78
Avg. weight (g)	0.468	34.45	102.09	226.3

## Data Availability

Data is contained within the article. Further inquiries can be directed to the corresponding author.
